# Supplementary material for: Two peptides targeting endothelial receptors are internalized into murine brain endothelial cells
Source: PLoS One. 2021 Apr 2;16(4):e0249686. doi: 10.1371/journal.pone.0249686 (PMC8018780; doi:10.1371/journal.pone.0249686)
Supplement: S1 Fig — Confluent monolayers of bEnd.3 cells were grown on glass-bottom imaging dish and were stained for claudin-5, CD31 (PECAM-1), and ZO-1 tight and adherens junction proteins (green), and the nuclei were stained by Hoechst 33342 (blue). Scale bars: 10 μm. (PDF) [file pone.0249686.s001.pdf]

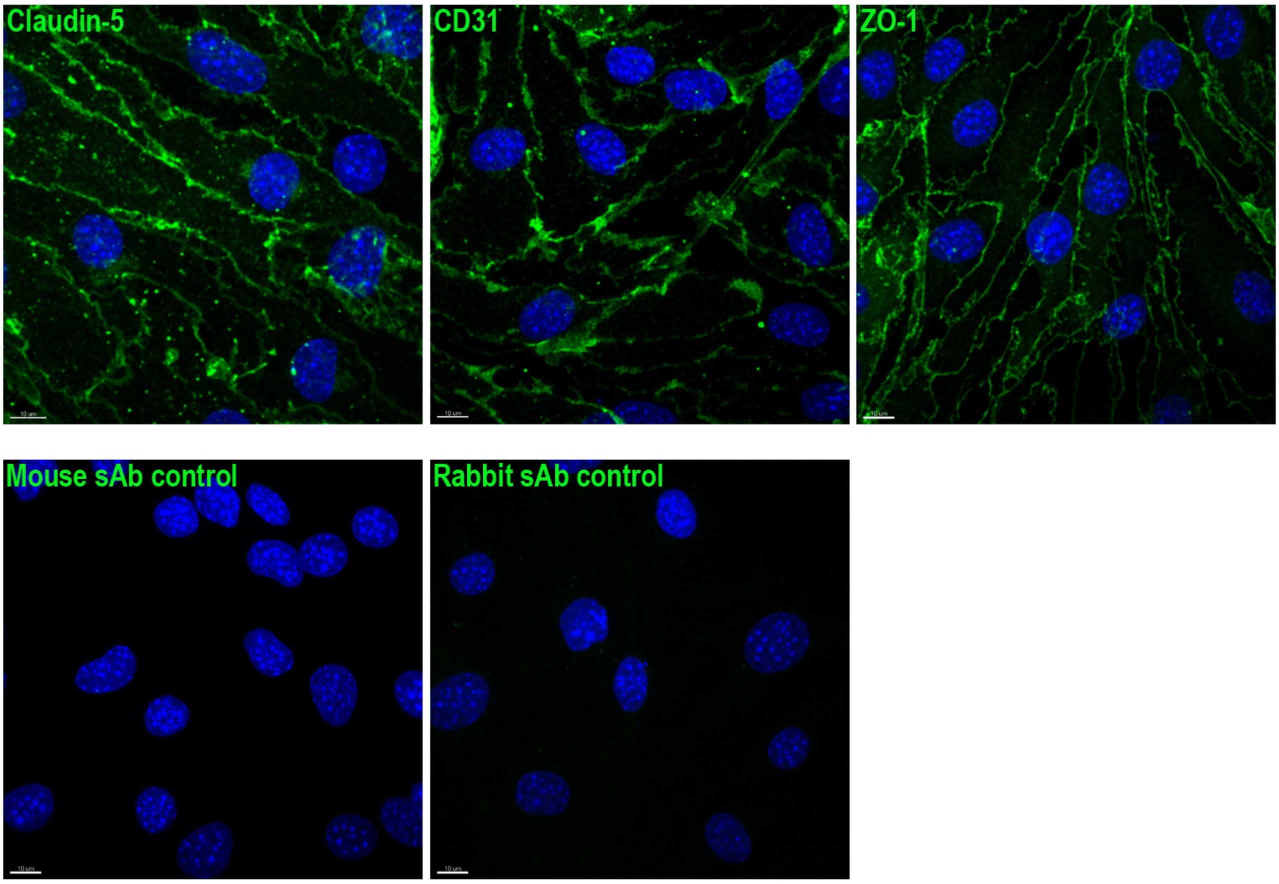


**S1 Fig. Expression of endothelial and BBB markers by bEnd.3 cells.** Confluent monolayers of bEnd.3 cells were grown on glass-bottom imaging dish and were stained for claudin-5, CD31 (PECAM-1), and ZO-1 tight and adherens junction proteins (green), and the nuclei were stained by Hoechst 33342 (blue).
Scale bars: 10 μm.
