# Supplementary material for: Two peptides targeting endothelial receptors are internalized into murine brain endothelial cells
Source: PLoS One. 2021 Apr 2;16(4):e0249686. doi: 10.1371/journal.pone.0249686 (PMC8018780; doi:10.1371/journal.pone.0249686)
Supplement: S2 Fig — The confluent bEnd.3 cells were exposed 10 μM TAMRA-labeled MTfp (red) for 1 hour at 4°C, then the internalization of the MTfp was followed for 120 minutes (chase). Representative 2D micrographs and cross-sectional views of the regions with MTfp (yellow dashed rectangles) show that no peptide internalization happened even after 2 hours chase. MTfp was only found on the cell surface. Cells were labeled by WGA (green). Scale bars: 10 μm, image acquisition: 100x silicone immersion objective. (PDF) [file pone.0249686.s002.pdf]

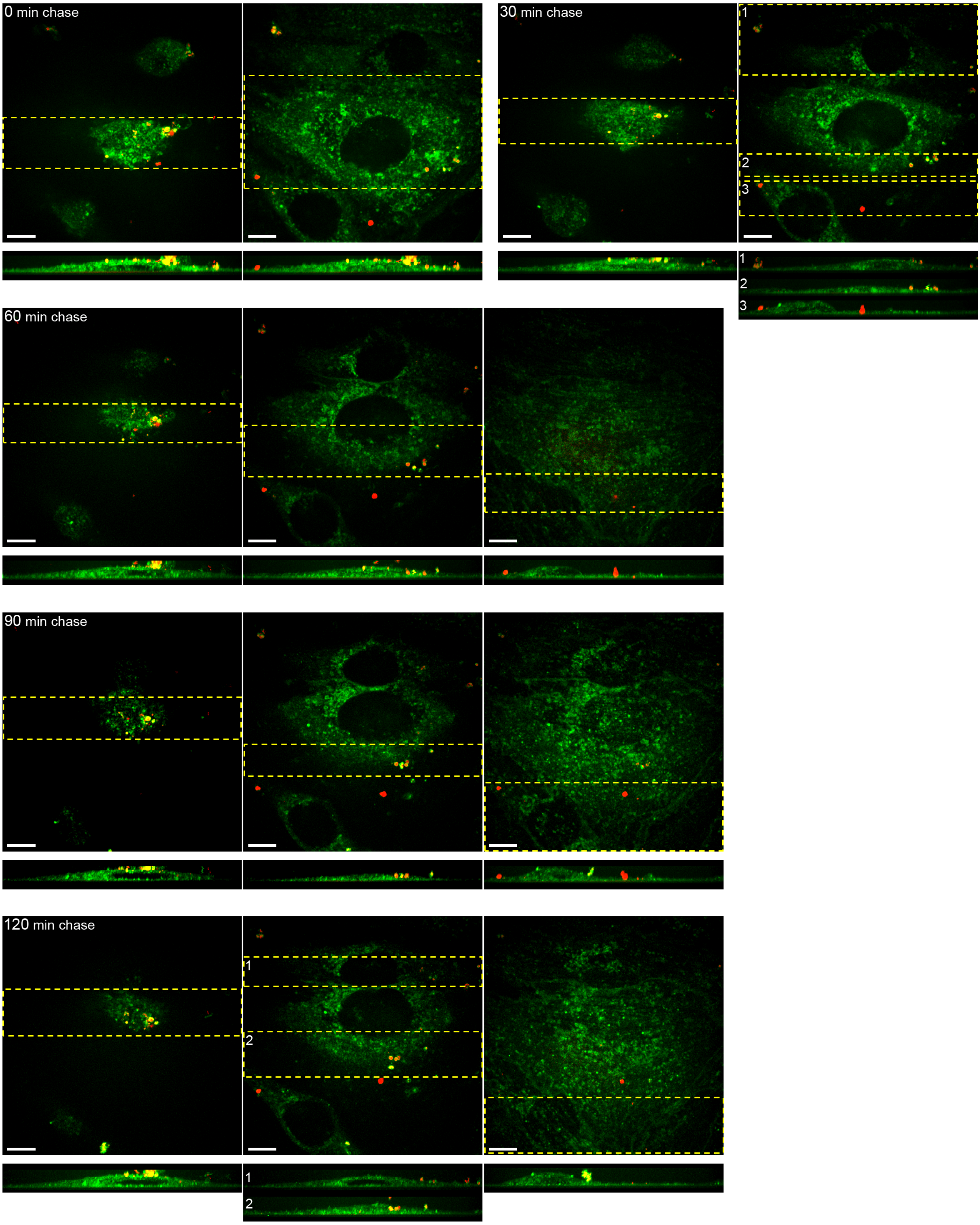

S2 Fig. Energy dependent uptake of TAMRA-labeled MTfp in confluent bEnd.3 cells.
The confluent bEnd.3 cells were exposed 10 μM TAMRA-labeled MTfp (red) for 1 hour at 4 °C, then the internalization of the MTfp was followed for 120 minutes (chase). Representative 2D micrographs and cross-sectional views of the regions with MTfp (yellow dashed rectangles) show that no peptide internalization happened even after 2 hours chase. MTfp was only found on the cell surface. Cells were labeled by WGA (green). Scale bars: 10 μm, image acquisition: 100x silicone immersion objective.
