# Supplementary material for: Two peptides targeting endothelial receptors are internalized into murine brain endothelial cells
Source: PLoS One. 2021 Apr 2;16(4):e0249686. doi: 10.1371/journal.pone.0249686 (PMC8018780; doi:10.1371/journal.pone.0249686)
Supplement: S3 Fig — The confluent bEnd.3 cells were exposed 10 μM TAMRA-labeled GYR peptide (red, circled with magenta) for 1 hour at 4°C, then the internalization of the peptide was followed for 120 minutes (chase). Representative 2D micrographs and cross-sectional views of the few regions with GYR peptide (yellow dashed rectangles) show that almost no peptide internalization happened even after 2 hours chase. Cells were labeled by WGA (green). Scale bars: 10 μm, image acquisition: 100x silicone immersion objective. (PDF) [file pone.0249686.s003.pdf]

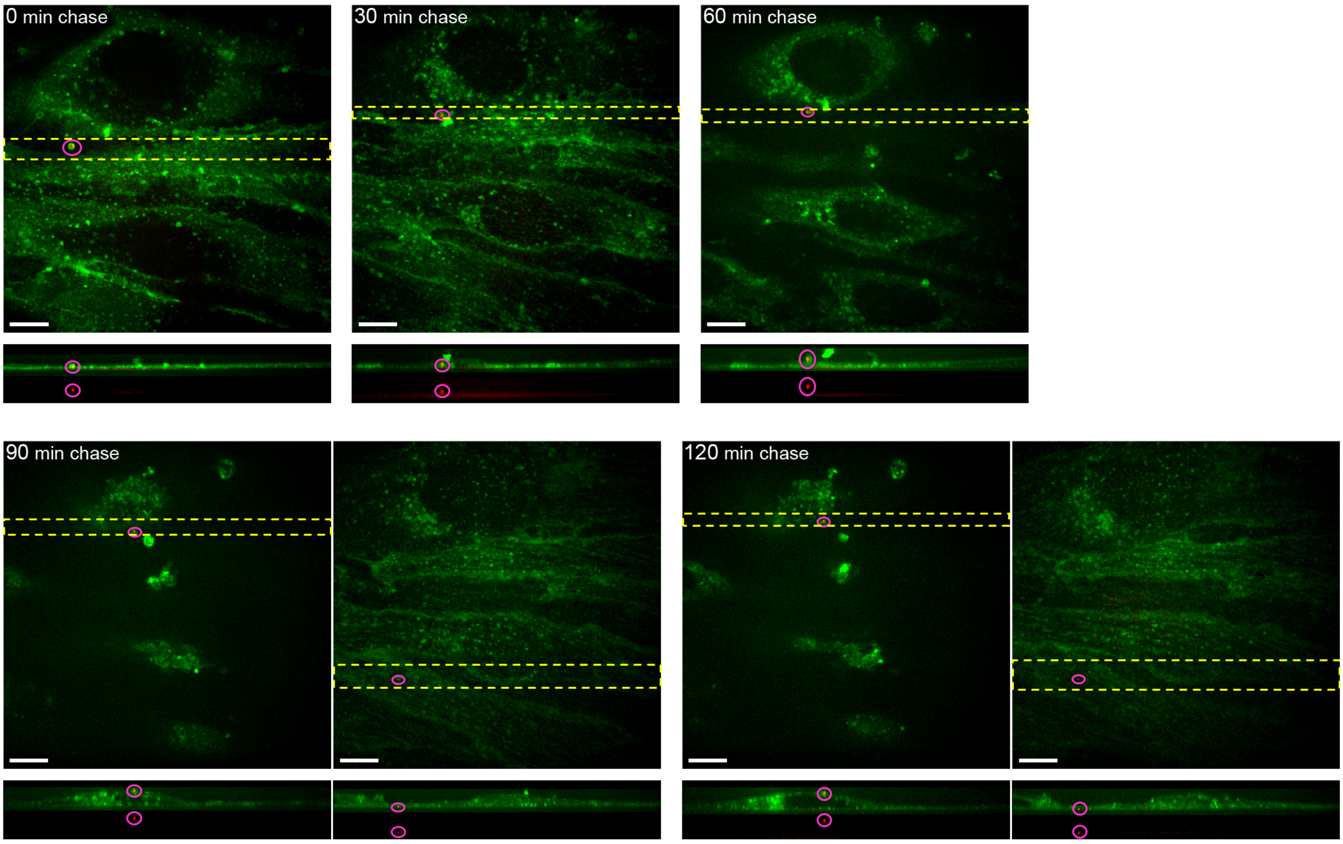

S3 Fig. Energy dependent uptake of TAMRA-labeled GYR peptide in confluent bEnd.3 cells.
The confluent bEnd.3 cells were exposed 10 μM TAMRA-labeled GYR peptide (red, circled with magenta) for 1 hour at 4 °C, then the internalization of the peptide was followed for 120 minutes (chase). Representative 2D micrographs and cross-sectional views of the few regions with GYR peptide (yellow dashed rectangles) show that almost no peptide internalization happened even after 2 hours chase. Cells were labeled by WGA (green). Scale bars: 10 μm, image acquisition: 100x silicone immersion objective.
