# Supplementary material for: Two peptides targeting endothelial receptors are internalized into murine brain endothelial cells
Source: PLoS One. 2021 Apr 2;16(4):e0249686. doi: 10.1371/journal.pone.0249686 (PMC8018780; doi:10.1371/journal.pone.0249686)
Supplement: S4 Fig — Representative maximum intensity projection images and cross sectional views of the highlighted sections (yellow rectangle) show the distribution of TfR (a) and LRP-1 (b) receptors (green) on bEnd.3 cell surface. Scale bars: 10 μm, image acquisition: 100x silicone immersion objective. (PDF) [file pone.0249686.s004.pdf]

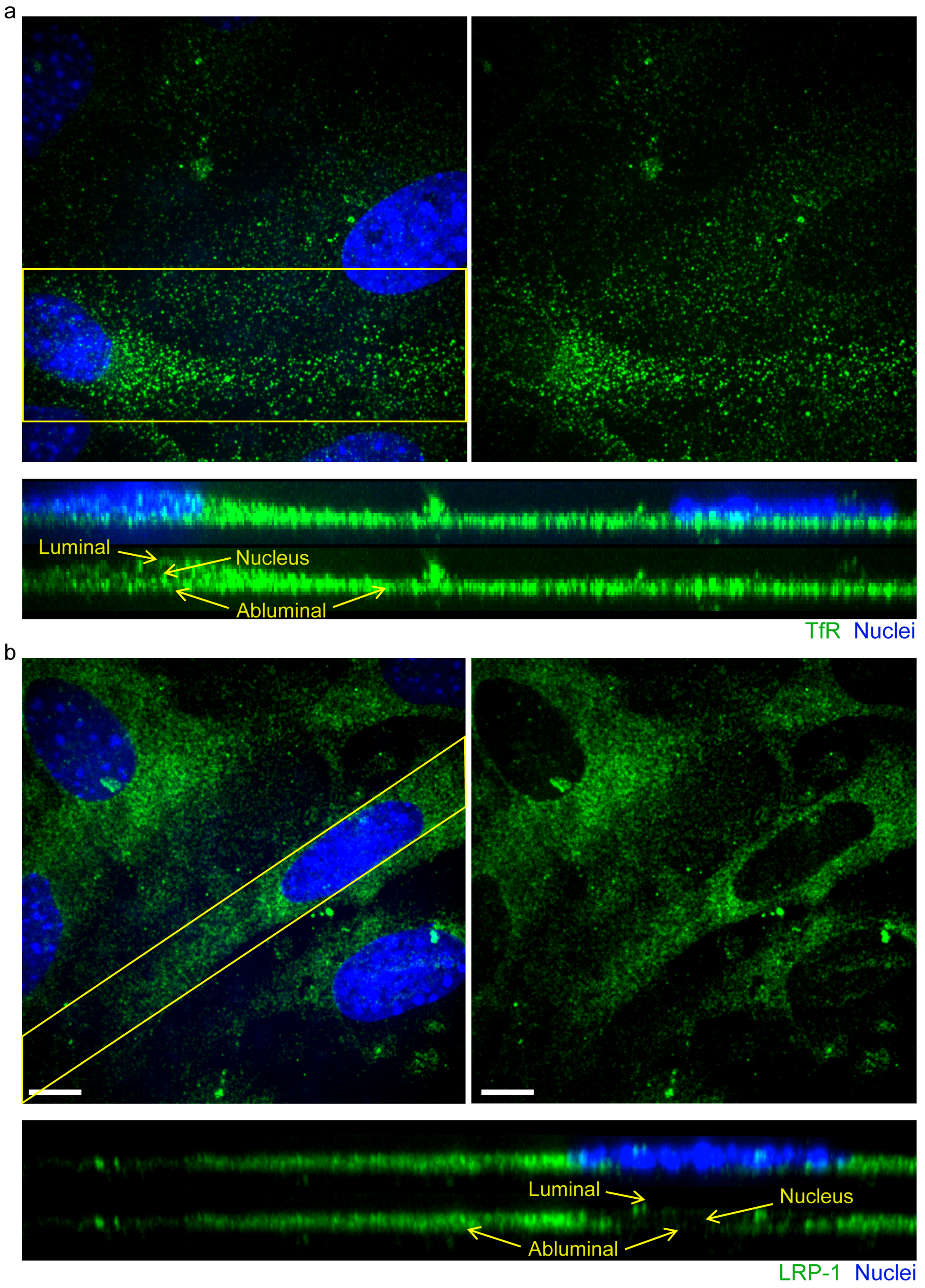

S4 Fig. Localization of TfR and LRP-1 receptors on bEnd.3 cell surface. Representative maximum intensity projection images and cross sectional views of the highlighted sections (yellow rectangle) show the distribution of TfR (a) and LRP-1 (b) receptors (green) on bEnd.3 cell surface. Scale bars: 10 μm, image acquisition: 100x silicone immersion objective.
