# Supplementary material for: Two peptides targeting endothelial receptors are internalized into murine brain endothelial cells
Source: PLoS One. 2021 Apr 2;16(4):e0249686. doi: 10.1371/journal.pone.0249686 (PMC8018780; doi:10.1371/journal.pone.0249686)
Supplement: S5 Fig — Transcytosis of 10 μM TAMRA-labeled MTfp (a) and GYR (b) across a coated cell-free polyester Transwell membrane. (a-b) Representative 2D micrographs of the top and bottom side of the membrane and crosssectional views show that both MTfp and GYR are trapped inside the pores (red: peptides). Scale bars: 20 μm. Image acquisition: 60x water immersion objective. The white dashed line marks the top part of the 10 μm thick membrane. (PDF) [file pone.0249686.s005.pdf]

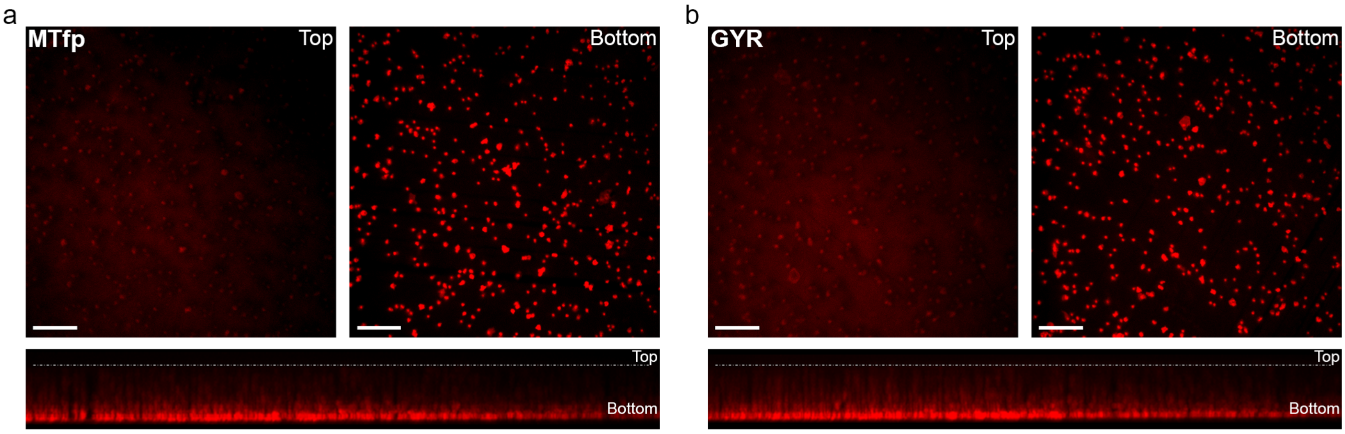

S5 Fig. Transcytosis control of MTfp (3) and GYRp (4).

Transcytosis of 10 μM TAMRA-labeled MTfp (a) and GYR (b) across a coated cell-free polyester Transwell membrane. (a-b) Representative 2D micrographs of the top and bottom side of the membrane and cross-sectional views show that both MTfp and GYR are trapped inside the pores (red: peptides).

Scale bars: 20 μm. Image acquisition: 60x water immersion objective. The white dashed line marks the top part of the 10 μm thick membrane.
