# Supplementary material for: Two peptides targeting endothelial receptors are internalized into murine brain endothelial cells
Source: PLoS One. 2021 Apr 2;16(4):e0249686. doi: 10.1371/journal.pone.0249686 (PMC8018780; doi:10.1371/journal.pone.0249686)
Supplement: Attachment — Submitted filename: Responses to Reviewers Comments.docx [file pone.0249686.s009.docx]

The manuscript has been greatly improved and many of the original concerns were addressed. A couple of requests remained to be answered, especially the calculation of the transcytosed peptide as a permeability coefficient, either as Papp or Pe. Another important point is the characterization of the model in terms of paracellular permeability using a marker molecule with the same MW as the peptide, eg. fluorsecently labele dextran. All the other points are minor that can be easily amended.

*Dear Editor and reviewers,*

*Thanks for the many nice comments and suggestions. We have now studied the permeability in more detail and evaluated the translocation using P_app_ and P_e_. As discussed by you and our comments in the last rebuttal, the bEnd.3 cells are not the best model to study transcytosis. Our major message in this publication is the binding and endocytosis of the peptides to endocytic receptors, which is the first step in a successful transcytosis from blood to brain. The transcytosis/translocation studies we have provided is indicative and should be further validated in vivo. This should hopefully be clear from the discussion and conclusion. Below we have addressed all concerns and comments point-by-point. We hope that the manuscript is acceptable now for publication in PlosOne.*

*Sincerely*

*Morten Nielsen*

Reviewer #1: Authors partially replied to my concerns, that has, from my opinion, improved the quality of the results and the manuscript. However, some parts can still be improved, in particular the transcytosis experiments :

- Authors claim for the MTFp that 103 % of the peptide are still in the apical compartment at the end of the experiment, whereas 3 % have transcytosed and that the rest is trapped in the coated insert. This kind of data is no really relevant and raise doubt about the methods and the quantification methods. I would suggest to the authors to use the clearance principle to calculate the MTFp permeability (Pe) across the cells. For this, I would suggest to refer to the method to generate a concentration-independent parameter as described by Siflinger-Birnboim et al. (A. Siflinger-Birnboim, P.J. Del Vecchio, J.A. Cooper, F.A. Blumenstock, J.M. Shepard, A.B. Malik, Molecular sieving characteristics of the cultured endothelial monolayer, J Cell Physiol, 132 (1987). 111-117. 10.1002/jcp.1041320115). At the end, with this method, the authors will be also able to calculate the % of recovery. Difference between the starting quantity of peptide and the remaining quantity will correspond to the part trapped in inserts and/or degraded by BEC. Because these peptides are also observed in lysosomes, this latter point might be then discussed in the manuscript. Authors are also encouraged to read this review on transport assessment when using in vitro BBB models : ”Santa-Maria AR, Heymans M, Walter FR, Culot M, Gosselet F, Deli MA, Neuhaus W. Transport Studies Using Blood-Brain Barrier In Vitro Models: A Critical Review and Guidelines. Handb Exp Pharmacol. 2020 Oct 11.”

*As suggested, the translocation was recalculated using the clearance principle and is presented as apparent and effective permeability (P_app_ and P_e_). To calculate the apparent permeability, we used the following equation based on Czupalla et al. (doi: 10.1007/978-1-4939-0320-7_34):*

$P_{app}\left[ \frac{cm}{s} \right]=\frac{B}{T}\cdot\frac{V_{b}}{A\cdot t\cdot60}$

*Where P_app_ is the apparent permeability, B is the relative fluorescence unit (RFU) at time t (120 min), T is the top chamber RFU at time 0 (assumed to be constant; hence, we used the top chamber RFU at 120 min), V_b_ is the volume of the bottom channel [ml][Kumar, #91], A is the cross-section area of the membrane [cm^2^], and t is the time [min]. The reason for using this equation instead of the suggested* $V=\frac{{[A]}_{b}\cdot V_{b}}{{[A]}_{t}} and P_{app}=\frac{dV}{dt\cdot A}$ *(where [A]_b_ is the concentration of the tracer in the basolateral compartment and [A]_t_ is the initial concentration of the tracer in the basolateral/top compartment) from Siflinger-Birnboim et al. (doi: 10.1002/jcp.1041320115) is that the former equation is entirely based on the measured fluorescence intensity and not on the calculated concentrations based on a standard curve, i.e. in our opinion, a slightly bit more accurate. Furthermore, we calculated the permeability coefficients using both equations and the results were very similar. In the case of GYR-TAMRA, the P_app_ were 6.64 x10^-6^ cm/s vs. 6.71 x10^-6^ cm/s vs. 6.31 x10^-6^ cm/s using RFU values, the calculated concentrations based on a standard curve or the calculated final basal concentration and the 10 µM initial apical concentration values, respectively. The new data has been included in the manuscript as Table 3.*

- Then, to justify that this model, in this condition, is suitable for permeability studies, authors should include a paracellular marker such as a 3kDa-Dextran control, with an almost similar molecular weight than the transcytosed peptides of interest. Then permeabilities of the peptides and dextran need to be compared together.

*We used 4 kDa Dextran (FD4 from Sigma, molecular weight (MW) is ranging between 3 and 5 kDa). Both P_app_ and P_e_ was calculated and included in the manuscript and the values were compared with each other. The P_app_ through the cell-free filter revealed that most of the MTfp are trapped inside the membrane (as it was confirmed by microscopy) as the P_app, filter, MTfp_ was smaller than P_app, filter, Dextran_. Since the MW of the labelled MTfp is smaller than the dextran (~2.6 kDa vs. ~4 kDa), the expected P_app, filter, MTfp_ is higher than P_app, filter, Dextran_ as it was seen in the case of GYR. Hence, Transwell membrane (or at least polyester membrane with 0.4 µm pore size) and fluorescence intensity-based analysis of the basolateral compartment are not suitable to measure the translocation of MTfp across the BBB. This part is included and explained in the manuscript as well.*

*All permeability values are presented in Table 3.*

- Authors wrote in the conclusion : “In conclusion, we have designed a platform allowing for the synthesis and testing of BBB-targeting peptides.”. I am not convinced that this is the main message of the manuscript and that this is really true. In addition, this kind of BBB-targeting system already exist because several labs around the world make this transcytosis or paracellular experiments, routinely, even with human BBB models that are often more tight and easy to handle than b.end3 cells.

*Thank you, we agree with you and it has been modified in the manuscript.*

Reviewer #2: The authors have addressed the main concerns pointed out in the review report. Thus, the quality of the manuscript increased substantiality. However, I still have some concerns related to the translocation assays and the respective statements.
The authors followed the reviewers’ suggestion and introduced a translocation assay. This is a big and important part of the work, since these peptides were designed to penetrate the brain. The model used is a co-culture, which is usually referred as an improvement to single cell models.
Major comments I would like to be addressed:
1. Was the integrity of barrier measured, using a fluorescent probe?

*The barrier integrity was determined by measuring the paracellular permeability of fluorescein isothiocyanate (FITC) labeled 4 kDa dextran and sodium fluorescein (376 Da) and these data are now included in the manuscript.*

2. Why did you use microscopy to detect translocation? You could have detect fluorescence in the basolateral compartment by measuring the fluorescence intensity of labeled-peptides.

*We used microscopy and traditional fluorescence intensity-based techniques to detect translocation. We used microscopy as we wanted to see if the peptides were able to be endocytosed and translocate through the membrane and taken up by the astrocytes grown in juxtaposition on the opposite side of the Transwell membrane. Binding to endocytic receptors is a critical point for further success in in vivo translocation and successful drug delivery. Although we did not observe any translocated peptides inside the astrocytes using microscopy (most of them trapped inside the pores of the polyester membrane), a degree of translocation was observed by fluorescence intensity measurement of the basolateral compartment.*

3. You should check stability of the peptides. Are peptides degraded in acidic conditions?

*This is a valid request. We initially labeled a large fraction of peptides with TAMRA, which subsequently was purified and validated using HPLC in smaller portions when needed. The purification over time did not indicate any degradation but we can unfortunately not provide any quantitative data.*

4. Can 2.9 and 3.7% translocation be considered good translocation? Have you determine a threshold for translocation in your BBB models? In my view, the authors cannot state that MTfp and GYR “transcytose across the BBB”. The authors should rephrase the following sentences:
Line 31 – “Moreover, our in vitro Transwell transcytosis experiments confirmed that both MTfp and GYR peptides were able to transcytose across a murine barrier. Thus, despite binding to endocytic receptors with different affinities, both peptides are able to transcytose across the murine BECs.”
Line 398 – “Our transcytosis studies suggest that both peptides can transcytose across the BBB.
*The translocation was recalculated and is now presented as apparent and effective permeability. These permeability values were compared with the permeability of 4 kDa dextran, and they are discussed in the manuscript.*

*We agree that we have made conclusions about transcytosis mechanisms that are not proved by our data. Therefore, the term “trancytosis” is now corrected to “translocation” in all relevant places in the manuscript text.*

5. What do the authors mean with “The rest were most likely trapped inside the membranes.”? Since the results are “2.9±0.8% MTfp and 3.7±0.2% GYR were able to transcytose into the basolateral compartment, whereas 103.1±2.6% and 94.6±0.5% remained inside the apical compartment” is there something left?
*As our translocation data is now presented as permeability (see Table 3), and the referred texts are now removed from the text.*

*However, we meant that in the case of the GYR, we collected 3.7 + 94.6 = 98.3% < 100%, and the remaining 1.7% is most likely trapped inside the filter membrane or in the endothelial cell layer. Again, we would like to stress that the receptor identification and binding is the most important message of the manuscript. Therefore the conclusion on translocation is less strong at the moment.*

Minor comments:
Line 20 – conjugating instead of attaching
Line 56 – I believe reference 21 is not correct, as Demeule et al 2002 does not use doxorubicin. Please check all references.
Line 182 – lacks a for five minutes
Line 224 – Lacks a “(“

*Many thanks for these observations, they are now corrected in the text.*

Reviewer #3: The authors have adequately addressed my comments and added several new data to the manuscript. They tested both the temperature dependency of the cellular uptake and the transcytosis of the peptides upon my request.
I have only one minor finding: the transcytosis of MTfp and GYR peptides would be better to show with the calculation of the apparent permeability coefficient and not with the form of % of transcytosed peptide.

*Thank you very much. The translocation of the peptides is now presented as apparent permeability coefficient. Furthermore, the calculated effective permeability coefficient values were also calculated (see Table 3).*

*Furthermore, following the statistical analysis guidelines of PLOS One, our data is presented as mean (SD) instead of mean ± SD. Furthermore, the normality of data distribution was assessed with GraphPad Prism 9.0; the QQ plots can be found below. These plots are not included in the manuscript.*
