# Supplementary material for: Two peptides targeting endothelial receptors are internalized into murine brain endothelial cells
Source: PLoS One. 2021 Apr 2;16(4):e0249686. doi: 10.1371/journal.pone.0249686 (PMC8018780; doi:10.1371/journal.pone.0249686)
Supplement: Attachment — Submitted filename: PONE-D-20-31660_ Reviewer Comments to authors.docx [file pone.0249686.s006.docx]

In “*Two peptides targeting endothelial receptors are internalized into murine brain endothelial cells*”, Sigurdardóttir et al. describes the interaction of two transBBB peptides with endothelial receptors, as well as the internalization pathway followed by both peptides in brain endothelial cells. The work is well organized and clear. However, the manuscript needs extra experiments and clarifications:

Major general comments:

1. I believe the two peptides employed in this study are not clearly described, should be clear their relevance and what distinguishes the peptides from other in the literature that target the same receptors. Therefore, I would like to know more about the rationale behind the design of both peptides. I think that the authors should include information on previous results that support the inclusion of both peptides in the study, and current applications (if possible).
2. In the literature, there are numerous examples of the use of bEnd.3 cells in *in vitro* model of BBB to evaluate peptides translocation ability. The manuscript would benefit from those assays to prove the translocation of MTfp and GYRp? It would also be interesting to should the capability of these peptides to deliver a cargo.
3. Another concern with the experimental design is related to the use of a mouse cell line (bEnd.3) instead of a human endothelial cell line. In the manuscript the authors show that both peptides have a good binding affinity towards human receptors (LRP-1 and hTfR) and poor binding affinity towards mouse receptors (mTfR). So, based on these results and the increased interest of human models, why did you select the bEnd.3 as your cell line model?
4. Figure resolution should be improved. For instance, in figure 3 the text and sensorgrams are out of focus.
5. All references from results section are gone. **“Error! Reference source not found”.**

My major concern is related to the innovative aspects of the manuscript. At first, it seems that peptides are original or at least not tested for BBB interaction. However, GYRp was developed by phage isolation from the brain and MTfp was used to deliver antibodies in vivo, where biodistribution demonstrates delivery of MTfp-antibody into brain parenchyma. Both peptides were tested in mice, thus it would be pertinent to test their ability in BBB models of human brain endothelial cells. The SPR results do not add additional information, since authors state “This suggests that LRP-1 is not the main receptor facilitating the transcytosis of MTf to the brain in vivo”.

Other particular comments:

**Abstract (Page 2)**

Line 26 – Some issue raise above concerning the use of a murine model if both peptides have more affinity towards human receptors.

Line 30 – “frequently” is a subjective concept.

**Introduction (Page 3)**

The introduction presents the problem of crossing the BBB in a very clear way. It shows the need for innovative strategies and the authors try to address this problem by using two peptides.

Line 47 – For MTf a fair description is present. Nevertheless, I would like to know a little more about the physiological role of MTf.

Line 50 – The sentences are confusing.

Line 54 – Do you think that the interaction with BBB receptors might be compromised by the conjugation to small molecule drugs? Did you consider the use of conjugated peptides in the study?

Line 57 – “(…) MTf and MTf-derived peptides”, Why did you select MTfp among the other “promising as brain delivery agents?”

Line 59 – Poor description. The authors need to present GYR peptide in a more clear way.

Line 63 – “(…) of GYR and MTf-derived peptides (…)” is not a completely true sentence. The authors use one MTf-derived peptide, namely the MTfp.

**Materials and Methods (Page 4)**

Line 70 – It is not fluorophores. The authors only conjugated both peptide to one fluorophore (TAMRA).

Line 70 – Why did you conjugated the fluorophore in the N-terminus for one peptide and in the C-terminus for the other one?

Line 176 – “relevant secondary antibodies” …

Line 193 – Why did the authors incubated both peptides for 30 min?

Line 228 – The concentrations should be presented in the same way. There are differences between the text and the figure, for instance, (250 mM, 500 mM) and (0.25 uM, 0.5 uM).

**Results (Page 13)**

All references are absent.

Line 234-248 – are not results but methods.

Line 260 – Please comment the SPR sensorgrams for GYRp. Sensorgrams are not a typical binding curve with association, steady state and dissociation. The fast dissociation is typical of non-specific binding.

Line 255 – The authors mention the use of WB to confirm the TfR and LRP-1 expression in bEnd.3; however, they do not present the results. Since this section is basically based on the SPR results, and in these experiments the authors do not use cells, is important to present those results.

Line 271 – “on the lower (basal) cell membrane indicating transcytosis”- is not correct to say “transcytosis”. The term transcytosis means that the molecule to be transported is captured in vesicles on one side of the cell, drawn across the cell, and release on the other side. In the results presented there is no confirmation of release on the other side.

The authors should use other controls to demonstrate internalization, for instance, perform the experiment at 4 C.

**Discussion (Page 16)**

Line 300 – “(…) targeting brain diseases (…)” instead of “(…) targeting diseases (…)”.

Line 305 – A comment on the conjugation at the N- or C-terminus would be helpful to understand the difference if the authors considered it interesting.

Line 319 – With substantially less affinity. Comment on that in the discussion.

Line 321 – Why is this to be expected?

Line 331 – The authors introduce OX26 as a “control”; however, they do not explain the relevance of using it. An explanation would improve its impact.

Line 334 – If you consider that the affinity would be different, why did you not use fluorescent-labeled peptides instead of unlabeled ones?

Line 343 – “fixability”

**Conclusions:**

The authors claim they have developed a platform for synthesis and testing of brain-targeting peptides. Do the authors consider LRP1 and TfR brain specific receptors? Do results show strong evidence of targeting to these receptors?

“Information on receptor binding affinity and intra-cellular transport is often lacking” the authors should consider other methods to determine transport pathways. Labeling of a single compartment, such as lysosomes is not sufficient to determine “intra-cellular transport”.
