# Supplementary material for: Two peptides targeting endothelial receptors are internalized into murine brain endothelial cells
Source: PLoS One. 2021 Apr 2;16(4):e0249686. doi: 10.1371/journal.pone.0249686 (PMC8018780; doi:10.1371/journal.pone.0249686)
Supplement: Attachment — Submitted filename: PONE-D-20-31660R1_ Reviewer Comments to authors.docx [file pone.0249686.s008.docx]

***PONE-D-20-31660R1***

The authors have addressed the main concerns pointed out in the review report. Thus, the quality of the manuscript increased substantiality. However, I still have some concerns related to the translocation assays and the respective statements.

The authors followed the reviewers’ suggestion and introduced a translocation assay. This is a big and important part of the work, since these peptides were designed to penetrate the brain. The model used is a co-culture, which is usually referred as an improvement to single cell models.

**Major comments** I would like to be addressed:

1. Was the integrity of barrier measured, using a fluorescent probe?
2. Why did you use microscopy to detect translocation? You could have detect fluorescence in the basolateral compartment by measuring the fluorescence intensity of labeled-peptides.
3. You should check stability of the peptides. Are peptides degraded in acidic conditions?
4. Can 2.9 and 3.7% translocation be considered good translocation? Have you determine a threshold for translocation in your BBB models? In my view, the authors cannot state that MTfp and GYR “transcytose across the BBB”. The authors should rephrase the following sentences:

Line 31 – “Moreover, our in vitro Transwell transcytosis experiments confirmed that both MTfp and GYR peptides were able to transcytose across a murine barrier. Thus, despite binding to endocytic receptors with different affinities, both peptides are able to transcytose across the murine BECs.”

Line 398 – “Our transcytosis studies suggest that both peptides can transcytose across the BBB.

5. What do the authors mean with “The rest were most likely trapped inside the membranes.”? Since the results are “2.9±0.8% MTfp and 3.7±0.2% GYR were able to transcytose into the basolateral compartment, whereas 103.1±2.6% and 94.6±0.5% remained inside the apical compartment” is there something left?

**Minor comments:**

Line 20 – conjugating instead of attaching

Line 56 – I believe reference 21 is not correct, as Demeule et al 2002 does not use doxorubicin. Please check all references.

Line 182 – lacks a for five minutes

Line 224 – Lacks a “(“
